# Editorial: Tumor cell mechanosensitivity: molecular basis

**DOI:** 10.3389/fcell.2024.1484725

**Published:** 2024-09-06

**Authors:** Claudia Tanja Mierke, Xian Hu, Mingxi Yao, Kay Oliver Schink, Michael Sheetz

**Affiliations:** ^1^ Faculty of Physics and Earth Systems Science, Peter Debye Institute of Soft Matter Physics, Biological Physics Division, Leipzig University, Leipzig, Germany; ^2^ Center for Cancer Cell Reprogramming, Faculty of Medicine, University of Oslo, Oslo, Norway; ^3^ Department of Biosciences, University of Oslo, Oslo, Norway; ^4^ Mechanobiology Institute, National University of Singapore, Singapore, Singapore; ^5^ Department of Biomedical Engineering, Southern University of Science and Technology, Shenzhen, Guangdong, China; ^6^ Department of Molecular Medicine, Institute for Basic Medical Sciences, University of Oslo, Oslo, Norway; ^7^ Molecular Mechanomedicine Program, Biochemistry and Molecular Biology Department, University of Texas Medical Branch, Galveston, TX, United States

**Keywords:** mechanosensation, focal adhesions, mechanotransduction, actin binding proteins, tension, forces

The Research Topic entitled “*Tumor Cell Mechanosensitivity: Molecular Basis*” addresses fundamental aspects of mechanobiology with emphasis on the field of cancer development and its malignant progression. Physical signals, such as matrix stiffness, topology, solid stress, tension and strain or fluid shear stress in vessels or tissues from the microenvironment can be sensed by the cells and act as inducer of a biochemical response (Di et al., 2023; Kalli et al., 2023; Mierke, 2024). The sensing of the mechanical cues may differ between cancer cells and normal cells (Sheetz, 2019), making it obvious that mechanical cues are involved in multiple signaling pathways and may be suitable targets for cancer therapy.

The Research Topic includes eight articles that comprises three Reviews, two Mini-Reviews, two Original Reach Articles and one Brief Research Report. The reviews deal with the role of focal adhesion kinase (FAK) in clinical settings, the role of forces in immuno-engineering and cancer-associated fibroblast (CAF)-derived exosomal miRNAs acting in the control of cancer malignancy. The first Review by Zhang et al. presents the function of FAK in cancer progression and discusses whether it may serve as a tumor marker for several different cancer types, including colorectal, gastric, liver and lung cancers. The second Review by Yoon et al. discusses the immune engineering and its potential in cancer immunotherapy whereby the emphasis is placed on the mechanobiological aspects of immune cells, such as T-cells, NK cells and macrophages. Immune engineering investigations comprising Chimeric antigen receptor (CAR) expressing T cells, TCR-engineered T cell (TCR-T) immunotherapies, and the BiTE strategy promoting the interaction of cytotoxic T cells with cancer cells, and mechanobiological approaches involving magnetic nanoparticles producing heat in response to an alternating magnetic field to control the heat-sensitive protein (HSP) promoter system and ultrasound regulating mechanotransduction. The third Review by Guo et al. focusses on the effect of stromal cells, like CAFs, within the tumor microenvironment and in particular on CAF-derived exosomal miRNAs, which induce the malignant progression of cancer, due to immune modulation, growth, migration and invasion, epithelial-mesenchymal transition (EMT), and resistance to therapy. It provides an overview on the different types of miRNAs and their effect in various cancer types.

The two Mini-Reviews cover the effect of stiffness on a specific mechanotransducer and the mechanicals stimulation via blood flow facilitating a specific interference of cells. The first Mini-Review by Fujimoto and Nakazawa covers the function of Four-and-a-half LIM domains 2 (FHL2) acting as a mechanotransducer in focal adhesions (FAs), the actin cytoskeleton and the nucleus based on the mechanical cues of the microenvironment, such as stiffness. The second Mini-Review by Xu et al. addresses whether and how the blood flow activates the binding of von Willebrand factor (vWF) to platelets and subsequently, its interaction to other vWFs on endothelial cells. Both Mini-Reviews highlight the impact of the mechanical properties of the microenvironment on cells, such as stiffness or fluid flow-induced shear stress. Thereby, mechanical signals, such as stiffness, fulfill a regulatory role on the mechanotransducer FHL2 that acts at different cellular locations. Consistent with these findings, a novel mechanochemical feedback circuit was found in which force-driven FHL2 localization enhances hypercontractility (Seetharaman et al., 2024).

The first Original Research Article by Mao and Nakamura presents how force dependent conformational alterations enable the interaction of the actin crosslinking protein Filamin A (FLNA) with a new mechano-sensitive binding partner, La-related protein 4 (LARP4). FLNA interacts with LARP4 in the cleft formed by C and D strands of immunoglobulin-like repeat 21 (R21) in the presence of force, whereby specific mutation in FLNA impedes (e.g., F277A) the interaction. In the absence of force, this interaction is blocked by the A strand of R20 of LARP4. The second Original Research Article by Monserrat Vela-Alcántara et al. describes the analysis of the expression of neurophilin-1 (NRP-1) in cancer cells, such as cervical (HeLa) and prostate cancer cells (PC-3), and normal cells, such as HPrEC and HEK, in relation to the mechanical environment of the cells, such as stiffness. For example, when the microenvironmental stiffness is raised, the expression of NRP-1 is upregulated in a stiffness-dependent feedback circuit. The effect is more pronounced in cancer cells compared to normal cells. The Brief Research Report by Hu et al. relates the different cleavage of the focal adhesion protein Talin with the shape of FAs. Transformed cells, such as Meljuso, A375P, and A2058 melanoma cells, displayed higher Talin cleavage levels inside FAs compared to non-transformed cells, specifically the human foreskin fibroblast (HFF-1) cell line. In addition, growth tests showed that the decrease of calpain cleavage in Talin diminished the growth of the transformed cells.

There is still a need for further research focusing on the bidirectional role of the mechanical properties of the microenvironment on cancer cells and cancer-associated cells, such as stromal cells, endothelial cells and immune cells. Mechanically determined regulatory mechanisms that determine the localization of specific proteins at certain locations in the cell, such as the membrane, cytoskeleton, cell nucleus or in endosomes, could be of interest. The role of the exchange of cargos or membrane components by extracellular vesicles induced by mechanical modifications of the cell environment or cells appears to be important for understanding the interaction of cancer cells and immune or stromal cells and endothelial cells.

## References

[B1] Di X. Gao X. Peng L. Ai J. Jin X. Qi S. (2023). Cellular mechanotransduction in health and diseases: from molecular mechanism to therapeutic targets. Sig Transduct. Target Ther. 8, 282. 10.1038/s41392-023-01501-9 PMC10387486 37518181

[B2] Kalli M. Poskus M. D. Stylianopoulos T. Zervantonakis I. K. (2023). Beyond matrix stiffness: targeting force-induced cancer drug resistance. Trends Cancer 9, 937–954. 10.1016/j.trecan.2023.07.006 37558577 PMC10592424

[B3] Mierke C. T. (2024). Extracellular matrix cues regulate mechanosensing and mechanotransduction of cancer cells. Cells 13, 96. 10.3390/cells13010096 38201302 PMC10777970

[B4] Seetharaman S. Devany J. Kim H. R. Van Bodegraven E. Chmiel T. Tzu-Pin S. (2024). Mechanosensitive FHL2 tunes endothelial function. bioRxiv., 2024.06.16.599227. 10.1101/2024.06.16.599227 PMC13324624 42192127

[B5] Sheetz M. (2019). A tale of two states: normal and transformed, with and without rigidity sensing. Annu. Rev. Cell Dev. Biol. 35, 169–190. 10.1146/annurev-cellbio-100818-125227 31412209 PMC7474971

